# Deficient Myocardial Organization and Pathological Fibrosis in Fetal Aortic Stenosis—Association of Prenatal Ultrasound with Postmortem Histology

**DOI:** 10.3390/jcdd8100121

**Published:** 2021-09-28

**Authors:** Fleur Zwanenburg, Marco C. DeRuiter, Lambertus J. Wisse, Conny J. van Munsteren, Margot M. Bartelings, Marie-Jose Goumans, Arend D. J. Ten Harkel, Monique R. M. Jongbloed, Monique C. Haak

**Affiliations:** 1 Department of Obstetrics and Prenatal Diagnosis, Leiden University Medical Center, Albinusdreef 2, 2333 ZA Leiden, The Netherlands; f.zwanenburg@lumc.nl; 2 Department of Anatomy & Embryology, Leiden University Medical Center, Einthovenweg 20, 2333 ZC Leiden, The Netherlands; M.C.de_Ruiter@lumc.nl (M.C.D.); L.J.Wisse@lumc.nl (L.J.W.); J.C.van_Munsteren@lumc.nl (C.J.v.M.); M.M.Bartelings@lumc.nl (M.M.B.); m.r.m.Jongbloed@lumc.nl (M.R.M.J.); 3 Department of Cell & Chemical Biology, Leiden University Medical Center, Einthovenweg 20, 2333 ZC Leiden, The Netherlands; M.J.T.H.Goumans@lumc.nl; 4 Department of Pediatric Cardiology, Leiden University Medical Center, Albinusdreef 2, 2333 ZA Leiden, The Netherlands; A.D.J.ten_Harkel@lumc.nl; 5 Department of Cardiology, Leiden University Medical Center, Albinusdreef 2, 2333 ZA Leiden, The Netherlands

**Keywords:** fetal aortic stenosis, prenatal ultrasound, postmortem histology, speckle tracking analysis, endocardial fibro-elastosis

## Abstract

In fetal aortic stenosis (AS), it remains challenging to predict left ventricular development over the course of pregnancy. Myocardial organization, differentiation and fibrosis could be potential biomarkers relevant for biventricular outcome. We present four cases of fetal AS with varying degrees of severity and associate myocardial deformation on fetal ultrasound with postmortem histopathological characteristics. During routine fetal echocardiography, speckle tracking recordings of the cardiac four-chamber view were performed to assess myocardial strain as parameter for myocardial deformation. After pregnancy termination, postmortem cardiac specimens were examined using immunohistochemical labeling (IHC) of key markers for myocardial organization, differentiation and fibrosis and compared to normal fetal hearts. Two cases with critical AS presented extremely decreased left ventricular (LV) strain on fetal ultrasound. IHC showed overt endocardial fibro-elastosis, which correlated with pathological fibrosis patterns in the myocardium and extremely disturbed cardiomyocyte organization. The LV in severe AS showed mildly reduced myocardial strain and less severe disorganization of the cardiomyocytes. In conclusion, the degree of reduction in myocardial deformation corresponded with high extent to the amount of pathological fibrosis patterns and cardiomyocyte disorganization. Myocardial deformation on fetal ultrasound seems to hold promise as a potential biomarker for left ventricular structural damage in AS.

## 1. Introduction

Fetal aortic stenosis (AS) is a rare congenital heart disease comprising 0.2–0.5 of 1000 live births. It is known for its wide spectrum of clinical severity and difficulty to predict the clinical course in pregnancy and after birth. Even the mildest forms, characterized by only a subtle flow acceleration across the aortic valve at mid-gestation, may develop into a life-threatening critical AS at birth, carrying a high risk of morbidity and mortality [1,2]. Critical AS at mid-gestation causes left ventricular dysfunction due to elevated ventricular pressure, which in turn produces left ventricular dilatation and myocardial damage. Subsequently, blood flow through the left side of the heart diminishes, ultimately provoking progression to hypoplastic left heart syndrome (HLHS) at birth, a condition with an even worse prognosis [3,4,5,6]. Fetal aortic valvuloplasty has been introduced to relief the fetal critical AS in utero in order to prevent progression to HLHS by enhancing recovery of left ventricular function and flow patterns [7,8,9]. Although promising, a technically successful fetal aortic valvuloplasty still carries a 50% risk of developing into an univentricular heart [10]. Independent of prenatal treatment, it remains difficult to predict the left ventricular development in time. Possible predictors of biventricular outcome have been described previously, but a conclusive diagnostic ultrasound parameter is still lacking [11,12,13].

Mahtab et al. demonstrated that borderline left ventricles (BLV) with similar macroscopic appearances can show great variety in microscopic ventricular organization and differentiation. In particular, a subset of BLV hearts resembled normal hearts, containing a well-organized cardiomyocyte alignment and proper organized intercalated disks with a normal expression pattern of important myocardial differentiation markers such as cardiac troponin-I, N-cadherin, and Connexin43 (Cx43). In contrast, the remaining BLV hearts presented histologic abnormalities similar to HLHS hearts, showing disturbed cardiomyocyte and intercalated disks organization along with an almost completely absent expression of myocardial differentiation markers. Ventricular organization and differentiation could therefore be a predictor of biventricular outcome [14]. In fetal AS, the degree of prenatal endocardial fibro-elastosis is also related to the possibility of a postnatal biventricular circulation [15].

Normal myocardial organization and differentiation allows a coordinated contraction of the myocardium via effective cell–cell coupling, which is necessary for optimal cardiac function. Endocardial damage, such as endocardial fibro-elastosis, also affects myocardial contraction. A non-invasive tool to assess myocardial wall motion in vivo comprises the novel echocardiographic technique speckle tracking. Speckle tracking quantifies the deformation of the myocardial tissue by following the motion of certain myocardial regions, the so-called speckles, over sequential ultrasound frames [16].

We hypothesized that ultrasonographic myocardial deformation corresponds with the level of myocardial organization, differentiation and fibrosis and is therefore an in vivo marker of ventricular damage in fetal AS. Studies assessing the correlation between prenatal imaging and histology are extremely rare. Here, we present four cases of aortic stenosis diagnosed in utero with varying degrees of severity and compare the myocardial deformation based on prenatal ultrasound to postmortem histopathologic characteristics.

## 2. Materials and Methods

This study was performed in accordance with the Dutch regulation for the proper use of human tissue for medical research purposes and conducted according to the principles of the Declaration of Helsinki (version 7, October 2013) and Dutch Fetal Tissue Act. The protocol was approved by the local Ethics Committee (NL66978.058.18).

**Figure 1:**
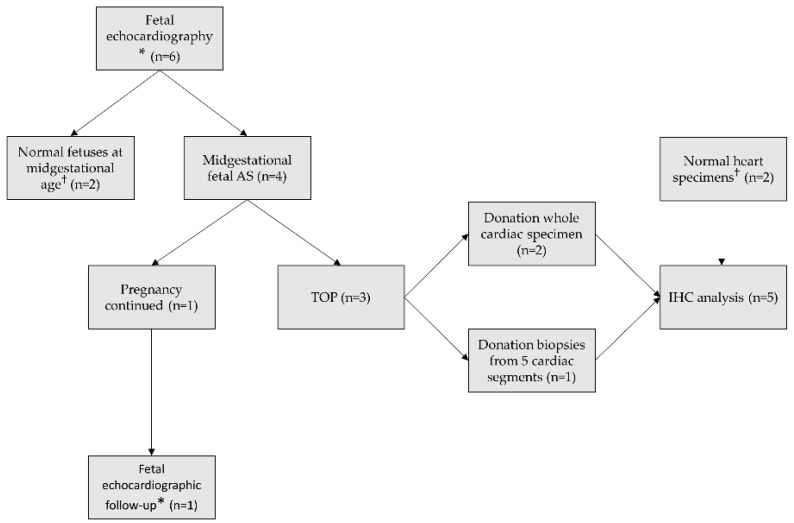
Overview flowchart of methods. * Offline speckle tracking analysis included; ^†^ Served as control data; AS, aortic stenosis; TOP, termination of pregnancy; IHC immunohistochemical.

An overview flowchart of the methods is depicted in Figure 1. We included cases with a midgestational diagnosis of fetal AS who presented at our tertiary fetal cardiology service between January 2018 and January 2020. Critical AS cases were defined as eligible for fetal aortic valvuloplasty based on the criteria described by McElhinney et al.: a valvular AS with an antegrade doppler color flow jet across the aortic valve which is smaller than the valve annulus diameter, no/minimal subvalvular LV outflow tract obstruction, decreased LV function with either retrograde/bidirectional flow in the transverse aortic arch at any time during the cardiac cycle or two of the following: monophasic mitral valve (MV) inflow, left-to-right flow across the atrial septum, bidirectional flow in the pulmonary veins [11]. Severe AS was defined as valvular AS with severely increased flow velocity over the aortic valve but with preserved left ventricular function and morphology of LV and thus not eligible for fetal aortic valvuloplasty at that time. To compare speckle tracking analysis with normal fetuses, we performed speckle tracking recordings in 2 healthy fetuses at midgestational age as well, of which informed consent was provided (NL65087.058.18).

Written informed consent was received from the women to perform speckle tracking recordings during routine fetal echocardiograms. The recordings were analyzed offline, weeks after the initial examination and therefore did not influence clinical decision-making. All women who decided to terminate the pregnancy (*n* = 3) gave signed written informed consent to donate cardiac tissue for immunohistochemical analysis. Two women donated the entire heart specimen and one woman gave permission to obtain heart biopsies taken from five segments (left ventricular apex, left ventricular base, right ventricular apex, right ventricular base, interventricular septum). The whole cardiac specimens were fixated in formaldehyde followed by storage in ethanol until use and the cardiac biopsies were immediately embedded in paraffin according to standard protocols. Two normal heart specimens (one entire heart specimen and one left ventricle (LV)) of similar gestational age served as immunohistochemical control cases, obtained from legal abortion material.

**Figure 2:**
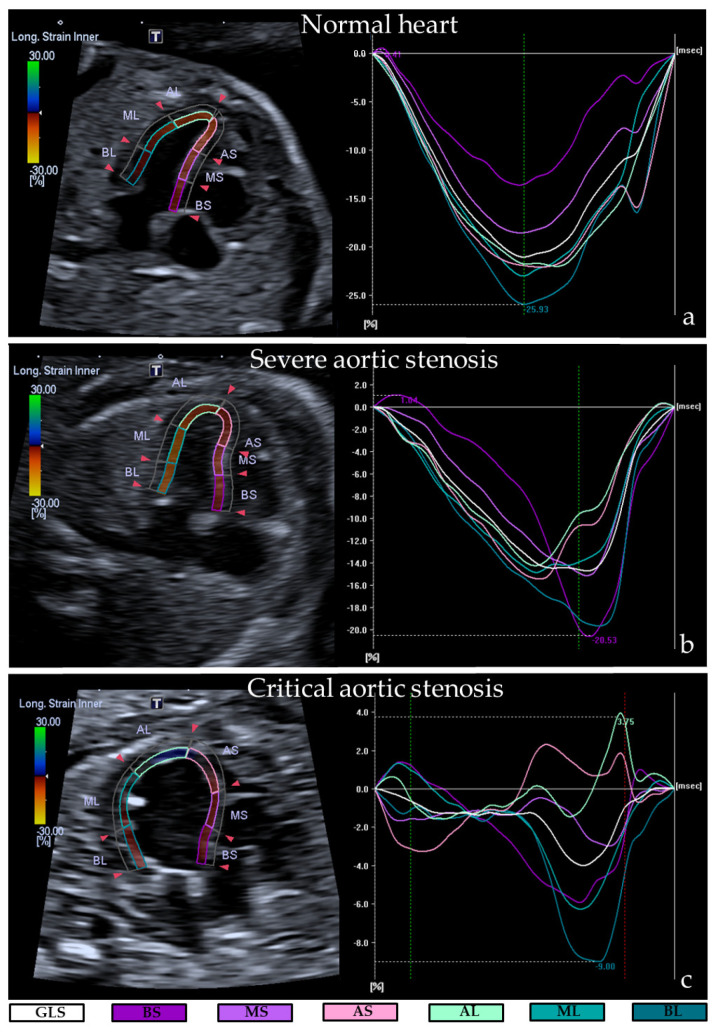
Decreased left ventricular myocardial deformation in cases with fetal aortic stenosis. Representative examples of global longitudinal strain and segmental longitudinal strain analysis for normal hearts (**a**), severe aortic stenosis (**b**) and critical aortic stenosis (**c**), showing the myocardial trace (**left**) and strain curves (**right**). Note that myocardial deformation is most overt in the critical aortic stenosis case. GLS, global longitudinal strain; BS, basal septal; MS, mid septal; AS, apical septal; AL, apical lateral; ML, mid lateral; BL, basal lateral.

*Echocardiography.* All fetal echocardiograms were performed by an experienced ultrasonographer (FZ) using the Canon Aplio i-800 ultrasound machine with abdominal PVI475BX and PVT674 High Frequency convex transducers. For speckle tracking recordings, cine loops of the apical or basal cardiac four chamber-view with an insonation angle of <30° were stored in B-mode setting. We optimized settings by adjusting sector width, depth, gain and zoom box to achieve framerates between 60–120 frames/s. Each clip contained at least 4 cardiac cycles in absence of maternal and fetal movements and was repeated 3 times. A measurement software package (Canon Medical Systems) was used for offline analysis; detailed methods of the analysis have been described previously [17]. In brief, speckle tracking analysis was performed on 3 different cardiac cycles for both the LV and right ventricle (RV), with the time cursor set between complete closure of the atrioventricular (AV)-valves and just before the next AV-valves closure. Endocardial markers were placed along the endocardium in end-diastole, after which the software automatically traced the inner and outer line of the endocardium. Global and segmental longitudinal strain of each ventricle (i.e., longitudinal shortening of entire ventricular wall and longitudinal shortening of separate segments of ventricular wall, expressed as proportion to the baseline length) were automatically calculated (Figure 2). Mean values of 3 cardiac cycles were used as definitive results.

*Immunohistochemical Analysis*. All cardiac specimens were embedded in paraffin according to standardized protocols. Frontal sections of 5 μm from whole hearts and 5 μm sections from the segmental biopsies were mounted on KP-Silane adhesive glass slides (Klinipath). Immunohistochemical and immunofluorescence staining was performed as described previously on the sections comprising the cardiac four chamber view and long-axis view (i.e., left ventricular outflow tract view) [14]. All used antibodies with the notation of their indication, dilution, species, clonality and source are outlined in Table 1. A cardiac troponin-I DAB (3-3′diaminobenzidine tetrahydrochloride) staining was performed to examine myocardial structure and organization. The presence of adherence junctions and gap junctions was assessed by double immunofluorescence staining with N-cadherin and Cx-43 using the suitable secondary antibodies Alexa Fluor 594-conjugated donkey anti-mouse IgG and Alexa Fluor 488-conjugated donkey anti-rabbit IgG. Cardiac fibrosis was identified using a Picro-Sirius Red staining (counterstained with Weigert’s Hematoxylin). For assessment of the epicardium, we used single DAB labeling with Wilms’ tumor 1 (WT-1), a marker for the epicardium and epicardial epithelial-to-mesenchymal transition (EMT). DAB staining for phosphorylated Smad2 (pSmad2) was used to examine EMT and endothelial-to-mesenchymal transition (EndMT), as both analogous processes are largely induced by the TGF-β/pSmad2 signaling pathway. EndMT was also assessed via immunofluorescence double staining of platelet endothelial cell adhesion molecule-1 (PECAM1) and α-smooth muscle actin (α-SMA) using the suitable secondary antibodies Alexa Fluor 555-conjugated donkey anti-rabbit IgG and Alexa Fluor 488-conjugated donkey anti-mouse IgG. Lastly, we examined the presence of subepicardial nerves using βIII-tubulin (TUBB3) DAB staining, a general nerve marker. High-resolution microscopy images of all glass slides were taken at 20× or 40× magnification with the 3DHistech Pannoramic 250 Flash III digital scanner or Leica SP8 confocal microscope. To examine the slides systematically, we studied the myocardial staining pattern in 4 predefined segments (LV basal, LV apical, septum and RV) and particularly in areas in which the cardiomyocytes were cut in their longitudinal axis.

## 3. Results

### 3.1. Case Description

Case 1 was diagnosed with a fetal critical AS at 20 + 3 weeks of gestation that met the international criteria for in utero aortic valvuloplasty (Table 2) [11]. The LV was spherical and presented poor contractility by eyeballing. After counseling, the parents opted for fetal aortic valvuloplasty one week later (21 + 2). The intervention was performed with a 2.7 mm-sized balloon inflated at 14 atmosphere. Immediately after balloon dilatation, aortic regurgitation and an increased amount of forward flow across the aortic valve was visualized. The valvuloplasty was complicated by hemopericardium with bradycardia, which resolved quickly through drainage of the hemopericardium. Three days after the procedure (21 + 5), the flow across the aortic valve had increased and the flow in the transverse aortic arch was antegrade, but the LV wall appeared thicker with no improvement in contractility. At 22 + 5, fetal echocardiography revealed LV shortening with poor contractility, scarce LV inflow and retrograde flow in the transverse aortic arch. The parents decided to terminate the pregnancy to avoid an univentricular trajectory for their child.

Case 2 presented at our fetal cardiology service at 19 + 5 weeks of gestation, revealing a critical AS with postvalvular dilatation (Table 2). The LV was enlarged and showed poor contractility. Genetic tests, comprising Quantitative Fluorescent Polymerase Chain Reaction (QF-PCR), array and whole exome sequencing (WES) were normal. Fetal aortic valvuloplasty was offered to the parents, yet they decided to withdraw from intrauterine treatment and proceeded to termination the pregnancy.

Case 3 presented with a severe fetal AS showing seriously increased flow velocity (350 cm/s) across the aortic valve at 20 + 1 weeks gestation. The international criteria for in utero aortic valvuloplasty were not met (Table 2) as the flow in the transverse aortic arch was antegrade and the LV appeared normal in shape and function. An amniocentesis was performed and results (QF-PCR and array) were normal. The parents feared progression to HLHS later in pregnancy and therefore, decided to terminate the pregnancy.

Case 4 was referred to our fetal cardiology service with an abnormal three vessel view, based on an enlarged aorta, at 21 + 1 weeks GA. We diagnosed a severe AS with an adequate size of the aortic annulus showing increased forward flow across the valve (300 cm/s) and postvalvular dilatation with turbulent flow (Table 2). As the LV was not dilated and there was normal antegrade flow in the aortic arch and biphasic inflow across the MV, this case was not eligible for fetal aortic valvuloplasty. Amniocentesis showed normal results for QF-PCR, array and WES. Extended echocardiography was performed every 2 weeks. The first signs of deterioration were present at 27 weeks GA as the LV was slightly shorter than the RV, showed less contractility and presented mild EFE. Moreover, monophasic inflow over the MV was present with little mitral insufficiency (MI). Although the criteria for intra-uterine balloon-plasty were met at that time, the annulus of the aortic valve was too big (5 mm) to perform an antenatal procedure. One week later (28 + 1), the flow in the aortic arch appeared to be retrograde in diastole and flow over the foramen ovale was bidirectional. Fortunately, the cardiac function stayed stable thereafter. A boy of 3730 g was born at 38 + 1 weeks GA. On the first day after birth, a percutaneous balloon aortic valvulotomy was performed with a 6 mm balloon. Bilateral pulmonary arterial banding followed at 8 days postpartum as a large ASD caused a substantial left-to-right shunt leading to pulmonary overcirculation. At 16 days postpartum, MV-plasty and closure of the atrial septal defect (ASD) with a fenestrated patch was performed. The LV function improved thereafter and debanding of the pulmonary arteries could be performed at 1 month postpartum. One year after birth, the child is doing well and develops normally.

### 3.2. Ultrasonographic Myocardial Deformation

Speckle tracking values of all cases are depicted in Table 3. Normal hearts showed a global longitudinal strain (GLS) and segmental longitudinal strain (SLS) between −15.5% and −22.9% for both LV and RV, which is in accordance with current literature [21]. The two cases with critical AS (case 1 and 2) presented extremely decreased left ventricular GLS and SLS values, indicating an impaired left ventricular myocardial wall deformation: mean left ventricular GLS was −2.0% and −0.9% and all mean left ventricular SLS were between −0.3% and −4.6%. The RVs showed an overall normal GLS (−19.0 and −16.0%) but the septal side, especially basal septal and mid septal, presented decreased SLS (between −4.0% and −15.3%). In contrast, the lateral sided SLS of the RV showed normal to slightly increased strain values in all cases (between −20.0% and −28.7%).

The two cases with severe AS (case 3 and 4) had normal LV appearance by eyeballing, but the left ventricular myocardial wall deformation showed a mild reduction (left ventricular GLS −11.8% and −10.5%, SLS between −5.3% and −16.0%). Right ventricular myocardial deformation was overall normal with a GLS of −17.6% and −21.2%. Again, in all severe AS cases the lateral side of the RVs showed slightly increased SLS values (SLS between −15.3% and 30.0%) and the septal side an overall mild reduction (SLS between −6% and −29.3%).

### 3.3. Histological Data

#### 3.3.1. Myocardial Organization and Differentiation

**Figure 3:**
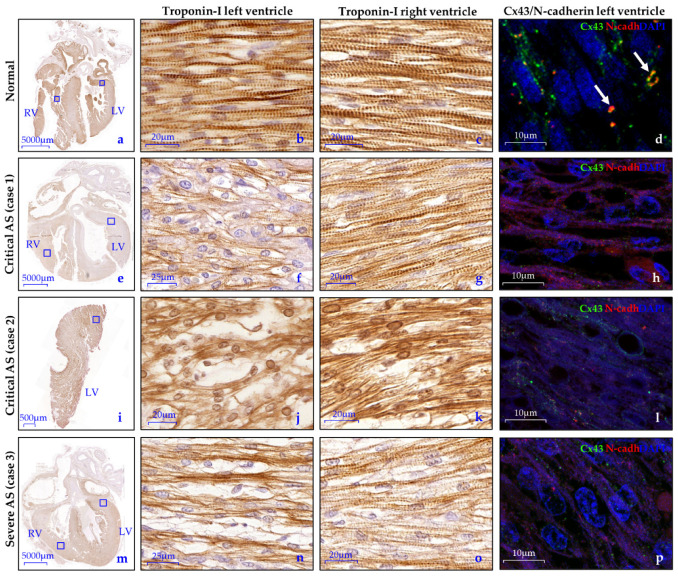
Disturbed alignment and altered Cx43/N-cadherin expression of the cardiomyocytes in fetal aortic stenosis. (**a**–**c**) In normal hearts, troponin-I staining shows an organized cardiomyocyte network with a proper banded pattern in the cardiomyocytes of both the LV and RV. (**d**) Cx43 (green) and N-cadherin (red) is found as a diffuse pattern in the cytoplasm as well as at the lateral borders of the cardiomyocytes, illustrating the presence of gap and adherence junctions. Some early intercalated disks are present, mostly expressing N-cadherin only but co-expression of N-cadherin and Cx43 is also seen in the intercalated disks (white arrows); (**e**–**p**) All AS hearts showed contrary findings; (**f**,**j**,**n**) The cardiomyocyte organization is disturbed in the LV with an increased amount of extracellular matrix, most apparent in case 1 and 2; (**h**,**l**,**p**) Cx43 and N-cadherin expression is markedly decreased and shows only a very scattered pattern in the cytoplasm with barely any lateralization; (**g**,**k**,**o**) The RVs of all specimens show regions with normal cardiomyocyte organization but also with mildly disturbed alignment. Negative controls are depicted in Supplementary Figure S1. Cx43, Connexin-43; *N*-cadh, N-cadherin.

In normal hearts, the cardiomyocytes of both the LV and RV were well-aligned and showed a proper banding pattern. Cx43 and N-cadherin were found as a diffuse pattern in the cytoplasm and were also expressed with more intensity at the lateral borders of the cardiomyocytes, illustrating the presence of gap and adherence junctions. Furthermore, some early intercalated disks were present at the longitudinal ends, mostly solely expressing N-cadherin but co-expression of N-cadherin and Cx43 was also found (Figure 3a–d).

In contrast, the LVs of the two cases with critical AS (case 1 and 2, Figure 3e–l) showed markedly disturbed cardiomyocyte organization and an almost completely absent banding pattern, as was visualized by troponin-I expression. Between the cardiomyocytes, the amount of extracellular matrix was increased as compared to the normal hearts (Figure 3f,j). Regional differences were not found. A less severely disturbed cardiomyocyte structure was observed in the LV of the severe AS case (case 3, Figure 3m–p). Interestingly, all AS cases showed severely reduced expression of Cx-43 and N-cadherin compared to the normal specimens. Their expression was only present as a very diffuse pattern in the cytoplasm with barely any lateralization and formation of intercalated disks, indicating a reduced amount of gap and adherence junctions (Figure 3d,h,l,p). All RVs showed regions with normal cardiomyocyte organization but also areas with mildly disturbed alignment. Expression profiles of Cx-43 and N-cadherin were identical to the LV in all cases.

#### 3.3.2. Endocardial and Myocardial Fibrosis Patterns

**Figure 4:**
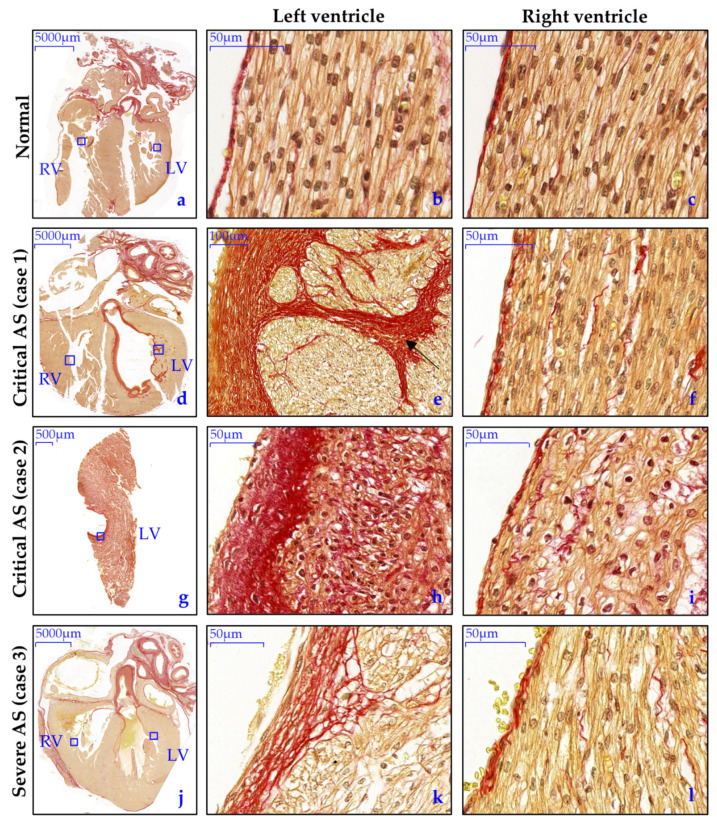
Overt endocardial fibro-elastosis with correlation to patchy fibrosis patterns in the myocardium in fetal AS. (**a**–**c**) In normal hearts, minimal and homogenous collagen deposition (red) is visible in the extracellular space between the cardiomyocytes and a thin layer of collagen is present in the endocardium; (**d**–**i**) the LV of the critical AS cases (case 1 and 2) shows overt EFE which is associated with patchy fibrosis patterns in the subendocardium an myocardium in case 1 (black arrow); (**j**–**l**) the severe AS case shows mild EFE with milder patchy fibrosis patterns in the subendocardium; (**c**,**f**,**i**,**l**) All RVs were similar to the normal LVs.

**Figure 5:**
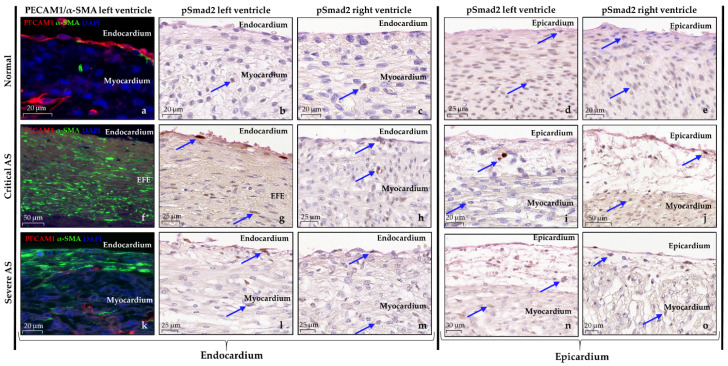
Altered expression markers of EndMT and EMT in cases with aortic stenosis. (**a**) In normal hearts, the endocardium consists of a flat layer of PECAM1 expressing endothelial cells (red) and a thin subendocardial connective tissue layer containing α-SMA positive cells (green). (**b**–**e**) Phospho-Smad2 (pSmad2) expression is rare at the endothelial site but highly present at the epicardial site; (**f**–**o**) Different expression profiles are seen in AS cases; (**f**,**k**) The EFE consists largely of α-SMA positive cells, representing activated fibroblasts. No PECAM1 positive endothelial cells are observed; (**g**,**h**,**l**,**m**) More expression of pSmad2 is found in the endothelial cells and cells in the EFE compared to normal hearts, which is similar between LV and RV; (**i**,**j**,**n**,**o**). At the epicardial site, the epicardium is thickened, rarely showing expression of pSmad2 in the LV of the critical AS cases while the LV of the severe AS specimen reveals a milder reduction in expression; (**j**,**o**) both RVs more closely resemble the normal hearts. PECAM1, platelet endothelial cell adhesion molecule-1; α-SMA, alpha smooth muscle actin; pSmad2, phosphorylated Smad2.

In normal hearts, Picro-Sirius Red (SR) staining exposed a homogenous pattern of interstitial fibroblasts, without signs of pathological fibrosis (Figure 4a–d). The endocardium consisted of a flat layer of PECAM1 positive endothelial cells (Figure 5a–c), and underneath a subendothelial layer containing collagen (highlighted by SR staining, Figure 4a–c) and α-SMA positive cells, presumably fibroblasts (Figure 5a). In the AS hearts, the degree and patterning of myocardial and endocardial fibrosis patterns were divergent and dependent on the AS severity. The critical AS heart specimens (case 1 and 2) showed a thick compact layer of endocardial fibro-elastosis in the LV which was remarkably associated with patchy pathological fibrosis patterns in the subendocardium and myocardium (Figure 4d,e,g,h). The severe AS specimen (case 3) only presented a thin layer of endocardial fibro-elastosis, with small patchy fibrosis patterns in the subepicardium and myocardium (Figure 4j,k). Right ventricular endocardial and myocardial SR staining was similar to the normal specimens in all AS cases (Figure 4c,f,i,l). EndMT has previously been suggested as underlying mechanism for endocardial fibro-elastosis development [22]. One of the key pathways that induces EndMT is the TGF-β signaling pathway via phosphorylation of Smad2 [23]. Therefore, we also determined the expression pattern of nuclear phosphorylated Smad2 (pSmad2). In normal hearts, the expression of nuclear pSmad2 is limited in the endocardium (Figure 5b,c). In contrast, in both the critical and severe AS cases, a subset of endothelial cells showed intense expression of nuclear pSmad2 and the nucleus of part of the cells in the endocardial fibro-elastosis also stained mildly positive (Figure 5g,h,l,m), indicative of activated TGF-β signaling. During EndMT, endothelial cells lose their expression of endothelial markers, such as PECAM1, and acquire mesenchymal characteristics such as expression of α-SMA [23]. In contrast to normal hearts, the endothelial surface in all our AS cases indeed did not contain PECAM1 positive endothelial cells but only α-SMA positive cells, which supports the occurrence of EndMT resulting in fully transformed endothelial cells (Figure 5f,k).

#### 3.3.3. Epicardium

**Figure 6:**
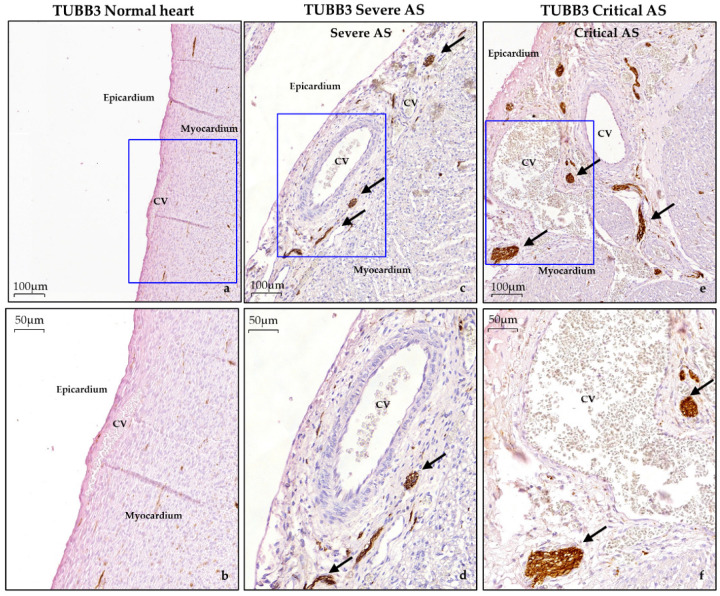
Abnormal epicardial thickening with dilated coronary vessels and large nerves in aortic stenosis cases. Representative images of the epicardium at two magnifications in: (**a**,**b**) normal hearts; (**c**,**d**) severe aortic stenosis; (**e**,**f**) critical aortic stenosis. Note the remarkable thickened epicardium containing dilated coronary vessels and large nerves (brown) in cases with aortic stenosis. TUBB3, βIII-tubulin; CV, coronary vessel.

Strikingly, in contrast to normal hearts, the subepicardium of both LV and RV was significantly thickened in all AS cases, suggesting epicardial activation. The subepicardium contained marked dilated coronary vessels which were surrounded by large TUBB3 positive nerves, while only a few nerve cells were seen in areas without vasculature (Figure 6). Next to EndMT, epicardial EMT has also been proposed as an underlying mechanism for endocardial fibro-elastosis development [24]. Unfortunately, staining of the widely used epicardial EMT and epicardial marker “WT-1” did not work on our material. EMT is largely induced by the TGF-β/pSmad2 pathway, similar to EndMT [25]. In normal hearts, in both LV and RV, nuclear pSmad2 was highly present at the epicardial site (Figure 5b–e). In contrast, the LV of the critical AS cases with the most severe endocardial fibro-elastosis demonstrated limited but more intense expression of nuclear pSmad2 in the subepicardial and myocardial cells, while the RV was similar to the normal heart specimens (Figure 5i–j). The severe AS specimen (case 3) revealed a milder reduction in nuclear pSmad2 expression at the epicardial site (Figure 5n–o).

## 4. Discussion

Fetal AS remains a challenging CHD considering its difficulty to predict left ventricular development over the course of the pregnancy and with that, the postnatal outcome. Myocardial organization, differentiation and fibrosis could be potential biomarkers, relevant for biventricular outcome [14]. The key finding of the current study is that fetal AS is associated with deficient myocardial organization, differentiation and pathological fibrosis patterns which were strongly associated with the prenatal ultrasonographic assessment of myocardial deformation (Table 4). Cx43 and N-cadherin expression, illustrating the presence of gap and adherence junctions, are altered in fetal AS cases independently of their severity. These results suggest that myocardial wall deformation on fetal ultrasound holds promise as a possible biomarker for myocardial organization and fibrosis and therewith represents the severity of left ventricular damage in cases with fetal AS. The severity of myocardial wall impairment seems unrelated to Cx43 and N-cadherin expression.

*Myocardial wall deformation in relation to histopathology.* In the current study, fetal AS cases with the most pronounced disturbance of the cardiomyocyte network and endocardial fibrosis patterns showed the utmost impairment of myocardial wall deformation on fetal ultrasound (Table 4). This is not surprising given that both cardiomyocyte disorganization and endocardial fibro-elastosis highly affect myocardial function. Inappropriate myocardial tissue arrangement impedes cardiac impulse propagation as well as coordinated myocardial contraction [26,27]. Likewise, endocardial fibro-elastosis is characterized by an excessive deposition of fibroblasts and accumulation of extracellular matrix, which leads to decreased ventricular diastolic compliance and impaired mechano-electric coupling of cardiomyocytes [24,28,29]. Previous studies in both adult human and animal models support the observed relationship. Adult patients with severe AS and still-preserved ejection fraction demonstrated a reduced left ventricular GLS which correlated to the severity of myocardial fibrosis [30,31]. Adult mouse models with isolated induced subendocardial fibrotic lesions or transverse aortic constriction showed that the reduction in longitudinal myocardial wall motion corresponded to histological myocardial changes [32,33]. In our study, even the case with mild disturbance of the cardiomyocyte network and endocardial fibrosis presented a corresponding mild reduction in myocardial wall deformation while, remarkably, standard LV function parameters according to the international criteria for fetal AS appeared normal. This emphasizes that speckle tracking echocardiography not only correlates greatly with myocardial histology but is also capable of identifying early structural alterations which are not detected by other ultrasound parameters [34]. The latter is especially of interest as Ishii et al. demonstrated slightly higher strain values before prenatal valvuloplasty in fetal critical AS cases with biventricular outcome compared to cases with univentricular outcome, suggesting a pivot role of speckle tracking analysis in predicting ventricular outcome at an early stage [35].

Although gap junctions and adherence junctions are important for the myocardial syncytium, the severity of myocardial dysfunction was not related to the degree of Cx43 and N-cadherin expression. In all AS cases, Cx43 and N-cadherin expression was mainly present in a very diffuse pattern in the cytoplasm with mild intensity. Vreeker et al. describes a similar expression profile in normal hearts at an earlier gestational age (approximately 15 weeks in utero), which changes gradually towards complete localization at the lateral borders and intercalated disks during prenatal and postnatal development [36]. One could hypothesize that AS hearts therefore have a delayed spatial development of Cx43 and N-cadherin. Another explanation could be a persisting altered expression, as electromechanical remodeling is known to occur in cases with left ventricular hypertrophy [37]. We demonstrated reduced Cx43 expression in all AS cases compared to normal hearts, which was independent of the severity of AS and left ventricular hypertrophy. This is in accordance with adult AS patients where similar expression levels were found between mild or severe ventricular hypertrophy [37], supporting previous reports that Cx43 is upregulated during the early stage of compensated hypertrophy followed by a downregulation in the chronic phase [38,39]. In contrast, Mahtab et al. only observed a Cx43 downregulation in the borderline left ventricles with extremely disturbed cardiomyocyte organization, while borderline left ventricles with proper cardiomyocyte organization showed normal expression levels [14]. In vitro experiments have shown that depletion of epicardial-derived cells (EPDCs) leads to downregulation of Cx43 and N-cadherin as well as dysregulation of the cardiomyocyte array [40]. An abnormal epicardial–myocardial interaction might be a possible explanation for the observed pathology.

*Pathophysiology of endocardial fibro-elastosis in aortic stenosis.* Altered hemodynamics in AS trigger the formation of endocardial fibro-elastosis via myocardial hypoxia [41,42]. In the current study we observed a typical endocardial fibro-elastosis appearance associated with patchy fibrosis patterns in the subendocardium and myocardium which was most overt in the critical AS cases. These “infiltrative” fibrosis patterns have been described previously in postnatal cases with HLHS but also in cases with flow disturbances across the aortic or mitral valve without the phenotype of HLHS and were demonstrated to increase with age [22]. This suggests that the degree of pathological fibrosis increases with longer and more severe exposure to altered ventricular blood flow and/or pressure, explaining the difference in amount of endocardial fibro-elastosis at similar gestational ages between our cases and also the known progressive character of this disease.

The origin of the accumulated fibroblasts in endocardial fibro-elastosis is still debated, where both endocardium-derived cells (EndMT) or epicardial-derived cells (EMT) have been proposed [22,24]. EndMT and EMT are comparable processes during normal cardiac development and comprise a subset of either endothelial or epicardial cells that transform into mesenchymal cells, induced by common biochemical pathways such as TGF-β/pSmad2 [25,43]. After embryonic development, the epicardium of adult patients with myocardial infarction is reactivated and regains fetal characteristics such as EMT [25], whereas EndMT is described in atherosclerosis, valvular disease and cardiac fibrosis [43]. In our study, the overt pathological thickening of the epicardium in all AS cases was suggestive of epicardial activation. Unfortunately, the EMT and epicardial marker “WT-1” did not work on our material to confirm this. Additionally, nuclear pSmad2 expression seems to be less expressed in the epicardium compared to normal hearts, which could indicate that the EMT theory is incorrect, but could also be related to a different time profile since pSmad2 expression is reduced towards the end of EMT/EndMT. Instead, pSmad2 was highly expressed in a subset of endocardial cells and in cells in the endocardial fibro-elastosis areas, although in limited number. Together with the absence of PECAM1 positive endothelial cells and the presence of α-SMA positive cells at the endocardial surface, this strengthens the theory that EndMT causes excessive myofibroblast deposition.

*Relation to development of HLHS.* Remarkably, the degree of cardiomyocyte disorganization in the critical AS cases was identical to the HLHS specimens described by Mahtab et al. [14], which could imply that these fetal AS cases are deemed to progress towards HLHS. The exact cascade for development from AS into HLHS is still not fully understood, although this is critically important for development of a targeted therapy in order to prevent an univentricular trajectory. Altered flow patterns and pressure overload undoubtedly are key triggers for the development of left ventricular hypoplasia. Endocardial fibro-elastosis also seems to play a crucial role in this process as fetal AS cases with more extensive endocardial fibro-elastosis on fetal ultrasound are more prone to an univentricular outcome [15]. Of note, the current study observed prominent endocardial fibro-elastosis in the critical AS cases with their myocardial histopathology resembling HLHS. The role of the epicardium and endocardium via EMT or EndMT needs to be further explored. An animal model which mimics fetal aortic stenosis would be beneficial to understand the pathway of AS into HLHS better, but no such model exists nowadays as it is hard to simulate, which emphasizes that fetal AS is a multifactorial disease.

*Right ventricular involvement in a left ventricular disease.* The RV has long been neglected in this typical left-sided heart disease. However, there is growing evidence that typical one-sided CHDs are in fact biventricular diseases [44]. The RV in all our AS cases presented also decreased SLS at the septal side, yet this is presumably attributable to the spherical shaped LV bulging into the RV due to the pressure overload resulting from the AS, as the SLS of the lateral side appeared normal to slightly increased. Of note, the current study showed, in accordance with Mahtab et al., altered Cx43 and N-cadherin expression in the RV of all AS cases which was identical to the LV. Whether this can be related to altered hemodynamics in the RV or to a common pathophysiological pathway remains unclear at this time.

## 5. Perspectives, Limitations and Conclusions

Although the current study material is unique and provides relevant insights in the pathophysiology of fetal AS, the number of studied specimens is too limited for statistical evidence. However, this case series shows that prenatal myocardial function associates to high extent with myocardial histology as the degree of the reduction in myocardial wall deformation on echocardiography corresponded with the histological amount of pathological fibrosis patterns and disorganization of the cardiomyocyte network. Therefore, fetal echocardiography, in particular GLS, seems to hold promise as a potential biomarker for structural myocardial damage. Current promising sophisticated genetic techniques, such as spatiotemporal single-cell RNA sequencing [45], may provide in-depth knowledge of genetic pathways involved in myocardial development and disease. Unraveling the underlying mechanisms and determinants of disease progression is essential to optimize counseling and to timely intervene to prevent a disease course towards univentricular physiology.

## Figures and Tables

**Table 1:** List of antibodies used for immunohistochemical and immunofluorescence analysis.

Primary Antibodies	Indication	Species	Clonality	Dilution	Source
Cardiac troponin-I	Myocardial structure	Rabbit	Polyclonal	1:1000	Abcam; AB47003
WT-1	Epicardial cells and EEMT	Rabbit	Monoclonal	1:200	Abcam; AB89901
TUBB3	Cardiac innervation	Rabbit	Polyclonal	1:8000	Sigma-Aldrich; T3952
N-cadherin *	Adherence junctions	Mouse	Monoclonal	1:200	Sigma-Aldrich; C3865
Cx43 *	Gap junctions	Rabbit	Polyclonal	1:600	Abcam; AB11370
PECAM1 *	Endothelial cells	Rabbit	Polyclonal	1:1000	Santa Cruz; sc1506R
α-SMA *	Activated cardiac fibroblasts	Mouse	Monoclonal	1:10,000	Sigma-Aldrich; A2547
pSmad2	Activated TGF-β signaling, specific for EndMT or EMT	Rabbit	Monoclonal	1:100	Cell Signaling; 138D4
**Secondary Antibodies**					
Antirabbit-Biotin Alexa Fluor 594 anti-mouse IgG Alexa Fluor 488 anti-rabbit IgG Alexa Fluor 488 anti-mouse IgG Alexa Fluor 555 anti-rabbit IgG		Goat Donkey Donkey Donkey Donkey	Polyclonal Polyclonal Polyclonal Polyclonal Polyclonal	1:200 1:200 1:200 1:200 1:200	Vector Laboratories; BA1000 Thermofisher Scientific; A21203 Thermofisher Scientific; A21206 Thermofisher Scientific; A21202 Thermofisher Scientific; A31572

* Stained with immunofluorescence. WT-1, Wilms’ tumor 1; eEMT, epicardial epithelial-to-mesenchymal transition; Cx-43, Connexin-43; TUBB3, βIII-tubulin; PECAM1, platelet endothelial cell adhesion molecule-1; α-SMA, alpha smooth muscle actin; pSmad2, phosphorylated Smad2.

**Table 2:** Ultrasonographic baseline characteristics at first presentation.

	Case 1	Case 2	Case 3	Case 4
GA (wks) first presentation	20 + 3	19 + 5	20 + 1	21 + 1
Diagnosis	Critical AS	Critical AS	Severe AS	Severe AS
Valvuloplasty	Yes, prenatal at 21 + 1 wks	Offered but declined by parents	No	Yes, postnatal
AoV diam (Percentile ^1^)	2.5 (P0)	2.4 (−1.940/−2.326)	2.5 (P1)	2.9 (P4)
AoV velocity cm/s	210	250	313	301
MV diam (Percentile ^2^)	4.6 (P4)	4.6 (P7)	4.8 (P10)	5.5 (P24)
TV diam (Percentile ^2^)	5 (P17)	4.8 (P18)	5.1 (P22)	5.6 (P31)
LV length (Percentile ^3^)	12.8 (P44)	15.4 (P66)	13 (P47)	15.2 (P53)
RV length (Percentile ^3^)	11.8 (P49)	12.3 (P57)	11.4 (P48)	14.5 (P62)
LV shape	Spherical	Spherical	Normal	Normal
Aortic arch flow	Retrograde	Retrograde	Forward	Forward
EFE	Moderate	Mild	None	None
MV flow	Monophasic, regurgitation	Minimal forward, monophasic, regurgitation	Biphasic, mild regurgitation	Biphasic, mild regurgitation
FO flow	Left–right	Left–right	Right–left	Right–left
PV flow	Normal	Normal	Normal	Normal
GA of TOP (wks)	23 + 1	21 + 2	21 + 2	N/A

All measurements are performed by in vivo ultrasonography. ^1^ Calculation percentile using reference values of Vigneswaran et al. [18]. ^2^ Calculation percentile using reference values of Schneider et al. [19]. ^3^ Calculation percentile using reference values of Tan et al. [20]. GA, gestational age; LV, left ventricle; RV, right ventricle; MV, mitral valve; TV, tricuspid valve; diam, diameter; AoV, aortic valve; EFE, endocardial fibro-elastosis; FO, foramen ovale; PV, pulmonary veins; TOP, termination of pregnancy; AS, aortic stenosis.

**Table 3:** Decreased myocardial deformation in aortic stenosis cases.

	Normal Hearts	Case 1	Case 2	Case 3	Case 4
FPS	68	115	65	60	63
**Left Ventricle**					
GLS	−19.6%	−2.0%	−0.9%	−11.8%	−10.5%
SLS—basal septal	−16.4%	−1.0%	−2.7%	−14.3%	−16.0%
SLS—mid septal	−18.8%	−0.6%	−1.0%	−13.7%	−13.3%
SLS—apical septal	−22.0%	−3.0%	−1.0%	−13.0%	−7.0%
SLS—basal lateral	−20.7%	−1.6%	−1.3%	−8.7%	−5.3%
SLS—mid lateral	−20.2%	−2.6%	−0.3%	−10.7%	−12.3%
SLS—apical lateral	−20.5%	−4.6%	−0.3%	−11.7 %	−11.7%
**Right Ventricle**					
GLS	−19.2%	−19.0%	−16.0%	−17.6%	−21.2%
SLS—basal septal	−15.5%	−4.0%	−4.0%	−9.7%	−6.0%
SLS—mid septal	−17.2%	−5.7%	−7.0%	−10.7%	−15.0%
SLS—apical septal	−18.5%	−15.3%	−14.7%	−12.7%	−29.3%
SLS—basal lateral	−20.9%	−28.7%	−20.0%	−26.3%	−25.0%
SLS—mid lateral	−22.9%	−26.0%	−24.0%	−26.3%	−23.7%
SLS—apical lateral	−18.9%	−27.3%	−24.3%	−15.3%	−30.0%

FPS, frame rate per second; GLS, global longitudinal strain; SLS, segmental longitudinal strain.

**Table 4:** Summary of main ultrasound and histological findings (left ventricle).

Modality	Indication	Normal Hearts	Severe AS	Critical AS
Fetal Echocardiography	Myocardial deformation	Normal strain between −15.5% and −22.9%	Mildly decreased strain between −7.0% and −16.0%	Extremely decreased strain between −0.3% and −4.6%
Histology	Myocardial organization	Well-organized cardiomyocyte alignment	Moderate disturbed cardiomyocyte organization	Severely disturbed cardiomyocyte network
Histology	Myocardial differentiation	Proper Cx43 and N-cadh expression at lateral borders and intercalated disks	Reduced Cx43 and N-cadherin expression, mainly as diffuse intracellular pattern	Reduced Cx43 and N-cadh expression, mainly as diffuse intracellular pattern
Histology	Myocardial fibrosis	No EFE	Mild EFE	Overt EFE with patchy fibrosis patterns in myocardium

## Data Availability

Data are available upon reasonable request.

## Supplementary Materials

The following are available online at https://www.mdpi.com/article/10.3390/jcdd8100121/s1, Supplementary Figure S1: Representative images of negative controls. (**a**–**d**) Representative images of Troponin-I and Cx43/N-cadherin expression and their negative controls in normal hearts, and (**e**–**h**) in aortic stenosis hearts. Cx-43, Connexin-43; N-cadh, N-cadherin.
