# Supplementary material for: Deficient Myocardial Organization and Pathological Fibrosis in Fetal Aortic Stenosis—Association of Prenatal Ultrasound with Postmortem Histology
Source: J Cardiovasc Dev Dis. 2021 Sep 28;8(10):121. doi: 10.3390/jcdd8100121 (PMC8540431; doi:10.3390/jcdd8100121)
Supplement: Supplementary file 1 [file jcdd-08-00121-s001.zip › jcdd-1352220-supplementary.pdf]

Supplementary data

Supplementary Figure S1. Representative images of negative controls.

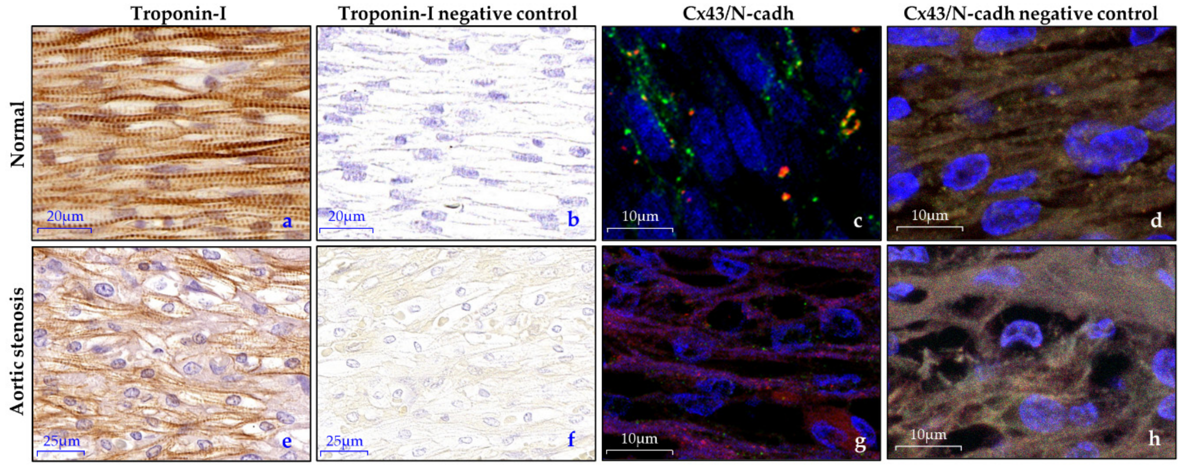

(**a-d**) Representative images of Troponin-I and Cx43/N-cadherin expression and their negative controls in normal hearts, and (**e-h**) in aortic stenosis hearts. Cx-43, Connexin-43; N-cadh, N-cadherin.
